# Adult‐onset epilepsy with startle‐induced seizure after febrile infection‐related epilepsy syndrome: A case report

**DOI:** 10.1002/epd2.70026

**Published:** 2025-04-15

**Authors:** Kazutoshi Konomatsu, Yosuke Kakisaka, Kazutaka Jin, Yu Fujiwara, Takafumi Kubota, Maimi Ogawa, Makoto Ishida, Kazushi Ukishiro, Hirohiko Ono, Kimihiko Kaneko, Naoto Sugeno, Masashi Aoki, Nobukazu Nakasato

**Affiliations:** ^1^ Department of Epileptology Tohoku University Graduate School of Medicine Sendai Miyagi Japan; ^2^ Department of Neurology Tohoku University Graduate School of Medicine Sendai Miyagi Japan; ^3^ Department of Psychiatry Self Defense Force Sendai Hospital Sendai Miyagi Japan

**Keywords:** adult‐onset epilepsy, case report, febrile infection‐related epilepsy syndrome, new‐onset refractory status epilepticus, reflex seizure, startle‐induced seizure

## Abstract

Startle‐induced seizure is a rare type of reflex seizure triggered by unexpected sensory stimuli that often occurs in children with early acquired cerebral lesions or brain malformations. We report a unique case of adult‐onset epilepsy with startle‐induced seizures. A 24‐year‐old woman had suffered high fever and focal to bilateral tonic–clonic seizures. A diagnosis of febrile infection‐related epilepsy syndrome (FIRES) was made based on the febrile infection occurring 7 days to 24 h before the onset of status epilepticus, which met all criteria for cryptogenic new‐onset refractory status epilepticus (NORSE) according to the cryptogenic NORSE score. Immunotherapy and several antiseizure medications resulted in transient resolution of the seizures. Four months later, she experienced startle‐induced seizures triggered by unexpected stimuli, such as auditory, visual, or unexpected events, and manifesting as initial tachycardia followed by right ear deafness, right hemifacial dysesthesia, eye deviation to the right, and tonic–clonic convulsions. Ictal electroencephalography revealed left temporal initial rhythmic delta activity, followed by rhythmic theta activity. The patient was diagnosed with startle epilepsy associated with FIRES and continued to receive anti‐seizure medications. Claustrum‐insular‐operculum lesions may have been the epileptic focus in this case, in contrast to previous cases of epilepsy with startle‐induced seizures originating in a frontoparietal network. This case indicates a new category of adult‐onset post‐FIRES epilepsy with startle‐induced seizures.

**Key points**

- A 24‐year‐old woman developed adult‐onset epilepsy with startle‐induced seizures after febrile infection‐related epilepsy syndrome.
- Seizures were triggered by unexpected stimuli, such as auditory, visual, or unexpected events.
- Seizures began with tachycardia followed by right ear deafness, right hemifacial dysesthesia, eye deviation to the right, and tonic‐clonic convulsions.
- Ictal EEG revealed left temporal initial rhythmic delta activity, followed by rhythmic theta activity.
- Claustrum‐insular‐operculum lesions may have been the epileptic focus, in contrast to previous cases of epilepsy with startle‐induced seizures originating in a frontoparietal network.

## 1 INTRODUCTION

Startle‐induced seizure is a rare type of reflex seizure, in which the seizures are triggered by unexpected sensory stimuli. Startle‐induced seizure may occur in patients with early acquired cerebral lesions, brain malformations, metabolic diseases, or chromosomal disorders. Typical seizures last <30 s and present as generalized seizures, such as tonic or atonic seizures, or focal seizures, such as hemitonic seizures.^1^ Childhood onset occurs in most patients, whereas only a few cases of adult onset are known.^2^, ^3^ Startle‐induced seizure typically involves the supplementary motor cortex, motor cortex, and premotor cortex.^1^, ^4^, ^5^, ^6^

New‐onset refractory status epilepticus (NORSE) is a clinical entity characterized by the new onset of refractory status epilepticus without a clear acute or active structural, toxic, or metabolic cause, in the absence of pre‐existing epilepsy or other relevant neurological disorders.^7^ Febrile infection‐related epilepsy syndrome (FIRES) is a subcategory of NORSE associated with the occurrence of febrile infection between 2 weeks and 24 h before the onset of refractory status epilepticus.^7^ Patients with FIRES often experience post‐FIRES epilepsy, but types of reflex seizure including startle‐induced seizure have never been reported in a patient with FIRES.

We present a case of adult‐onset epilepsy with startle‐induced seizures which occurred 4 months after the initial development of FIRES.

## 2 CASE REPORT

A 26‐year‐old woman was referred to our hospital for recurrent focal to bilateral tonic–clonic seizures (FBTCS). Her past medical history and family history were unremarkable, except for a previous episode of FIRES. She had developed a high fever and gastric discomfort at age 24 years, followed by repetitive FBTCS 4 days later. Diazepam and levetiracetam were administered, and the seizures were temporarily resolved, but her consciousness remained impaired. CSF analysis showed a mildly elevated white blood cell level but detected no infectious pathogens. Brain MRI showed no abnormalities on fluid‐attenuated inversion recovery (FLAIR) imaging and diffusion‐weighted imaging (DWI). However, arterial spin labeling imaging revealed hyperperfusion in the left temporal cortex. Short‐term scalp EEG revealed generalized rhythmic delta activity.

Her consciousness improved by day 2, but she suffered recurrent FBTCS with forced eye deviation to the right on day 5, followed by head version to the right and right‐leg clonic and bilateral tonic–clonic convulsions. These seizures were refractory to treatment with intravenous diazepam, levetiracetam, fosphenytoin, lacosamide, and midazolam. CSF analysis revealed a normal immunoglobulin G index, normal interleukin (IL)‐6 levels, and markedly elevated IL‐8 levels (68.8 pg/mL). However, negative findings were obtained for the oligoclonal band; autoantibodies in CSF, including N‐methyl‐D‐aspartate receptor, leucine‐rich glioma‐inactivated 1, contactin‐associated protein‐like 2, α‐amino‐3‐hydroxy‐5‐methyl‐4‐isoxazolepropionic acid receptor, γ‐aminobutyric acid type B receptor, glial fibrillary acidic protein, aquaporin 4, and myelin oligodendrocyte glycoprotein (MOG); and autoantibodies in the serum, including glutamic acid decarboxylase, aquaporin 4, and MOG. Short‐term scalp EEG revealed independent bilateral periodic delta activity on day 6.

**FIGURE 1:**
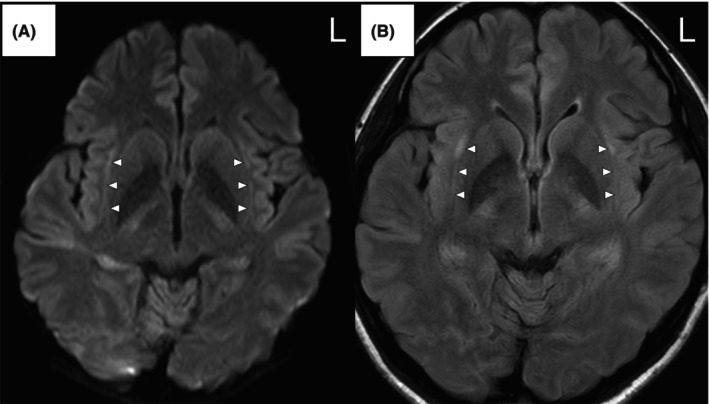
Brain MRI revealing high signal intensity on diffusion‐weighted (A) and fluid‐attenuated inversion recovery (B) imaging within the bilateral claustrum (arrowheads).

Autoimmune encephalitis was suspected, so high‐dose intravenous corticosteroids were administered on days 6–8 and again on days 15–17. Antiseizure medication adjustments included continued levetiracetam and lacosamide, with the addition of clobazam. Seizures ceased following corticosteroid treatment. Brain MRI on day 20 showed high signal intensity on DWI and FLAIR imaging (Figure 1A,B), and apparent diffusion coefficient mapping showed iso‐signal intensity within the bilateral claustrum. Interictal EEG revealed intermittent slow waves in the bilateral temporal regions.

A diagnosis of FIRES was made based on the febrile infection which had occurred within 7 days to 24 h before the onset of status epilepticus, meeting all six criteria for NORSE according to the cryptogenic NORSE score, consisting of: (1) NORSE highly resistant to conventional anti‐seizure medication treatments, (2) previously good health before the onset of status epilepticus, (3) presence of prodromal high fever of unknown origin, (4) absence of prodromal psychobehavioral or memory changes, (5) absence of sustained orofacial‐limb dyskinesias in an unresponsive state, and (6) DWI or T2‐weighted/FLAIR imaging findings of symmetric hyperintensities.^8^ The patient was discharged after 60 days with a medication regimen consisting of levetiracetam (3000 mg/day), lacosamide (300 mg/day), and clobazam (20 mg/day).

Four months later, the patient experienced spontaneous and startle‐induced seizures triggered by unexpected stimuli, such as auditory, visual, or news events during both awake and asleep periods. Startle‐induced seizures were provoked by loud noises from clashing dishes, the sudden appearance of a cat in an open‐air bath, seeing unexpected news on television, or even incidents within dreams. Both spontaneous and startle‐induced seizures began with tachycardia and a brief “generalized blood‐rushing” sensation followed in 20% of seizures by deafness (90% on the right, exceptional left‐sided dominance, or bilateral), then by dysesthesia of the right side of the face, occasionally accompanied by auditory hallucinations. Finally, forced eye deviation to the right and tonic–clonic seizures were observed. Seizures presenting with tachycardia and blood‐rushing sensation occurred weekly, whereas episodes involving deafness, dysesthesia, and FBTCS occurred 2–3 times per month. The patient was referred to our hospital for further evaluation.

**FIGURE 2:**
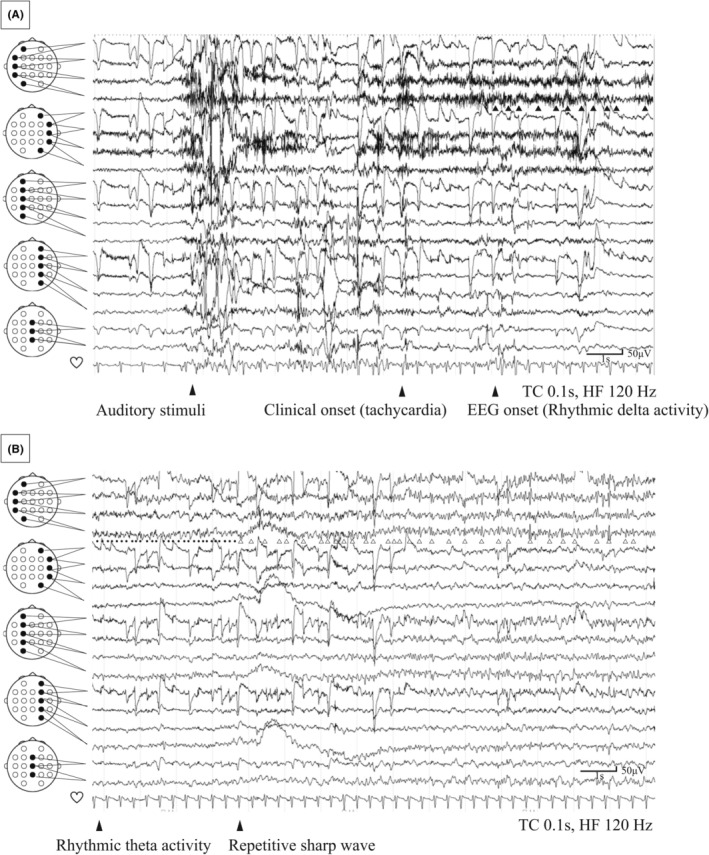
EEG revealing left temporal rhythmic delta activity (black arrowheads) 8 s after the auditory stimulus. The heart rate is 90/min before the seizure and increased to 156/min during the seizure (A). EEG revealing left temporal rhythmic theta activity (black dot) and repetitive sharp waves (white arrowheads) 35 s after the auditory stimulus (B).

Neurological examination and blood tests were unremarkable. Long‐term video EEG monitoring showed interictal spikes in the bilateral temporal regions. Habitual seizures were triggered by noise from the video monitor, loud sounds from nearby patients, or objects dropping in the room. Ictal EEG started with left temporal rhythmic delta activity, followed by rhythmic theta activity and repetitive sharp waves (Figure 2A,B). The heart rate was 90/min before the seizures and increased to 156/min during the seizures. Brain MRI showed no lesion on the bilateral claustrum, and fluoro‐2‐deoxy‐D‐glucose‐positron emission tomography revealed no abnormalities. The Wechsler Adult Intelligence Scale test showed normal intelligence. The diagnosis was post‐FIRES epilepsy with startle‐induced seizure. The addition of lamotrigine and reduction of lacosamide reduced seizure frequency. Over 16 months, the medication was adjusted to levetiracetam (3000 mg/day), clobazam (20 mg/day), and lamotrigine (400 mg/day). Spells of deafness and dysesthesia resolved, but weekly seizures with tachycardia and blood‐rushing sensations persisted, together with the monthly FBTCS episodes.

## 3 DISCUSSION

This unique case of adult‐onset post‐FIRES epilepsy with startle‐induced seizures triggered by various sensory stimuli demonstrates important differences from previously reported cases of epilepsy with startle‐induced seizures. These differences include the age of onset, etiology, seizure semiology, and EEG findings. Various conditions associated with startle‐induced seizures have been reported, including perinatal hypoxic injury, structural abnormalities such as cortical dysplasia and schizencephaly, metabolic encephalopathy, and chromosomal abnormalities.^1^, ^3^ However, epilepsy with startle‐induced seizures has never been reported in association with NORSE/FIRES. Adult‐onset NORSE was cryptogenic in 49.9% and autoimmune in 36.2% of previous cases.^9^ In the present patient, all autoantibody test results were negative, and no infectious causes were identified, leading to a diagnosis of cryptogenic FIRES. Typical startle‐induced seizures present as tonic, atonic, or hemitonic seizures.^1^, ^3^ However, this patient presented with tachycardia, ictal deafness, dysesthesia, and FBTCS. Previous studies have shown that ictal EEG in startle‐induced seizures usually includes an initial vertex discharge followed by diffuse relative flattening or low‐voltage rhythmic activity.^1^, ^2^ In contrast, the seizures in this patient started with left temporal rhythmic delta activity, followed by rhythmic theta activity and repetitive sharp waves.

Previous investigations have reported that the most common triggers of startle‐induced seizures are unexpected sensory stimuli, usually auditory stimuli.^1^, ^10^ Although the detailed mechanisms underlying startle‐induced seizures remain unclear, existing evidence suggests involvement of the frontoparietal network.^1^ In contrast, the startle‐induced seizures in the present case were triggered not only by auditory stimuli but also by visual stimuli and unexpected events. The mechanism by which the claustrum influences epileptic seizures is not well understood, but the involvement of multisensory stimuli as triggers in this case suggests the probable involvement of the claustrum. The claustrum is known to integrate multisensory signals and to participate in salience detection, which are processes closely related to the perception of startle stimuli.^11^ Recent functional MRI studies have demonstrated that the claustrum is functionally connected to the brain regions involved in salience processing, such as the insular cortex.^11^ While the claustrum sign is neither diagnostic nor specific for FIRES, it has been reported as a radiological marker of cytokine‐mediated neuroinflammation.^12^ Given these findings, we suggest that the claustrum–insular–operculum was probably involved in the startle‐induced seizure triggered by multisensory stimuli observed in this patient.

Symptomatogenic zones for autonomic seizures may include the insular cortex, cingulate gyrus, orbitofrontal cortex, and mesial temporal structures.^13^ In the present case, a claustral lesion adjacent to the insular cortex was observed, suggesting an insular cortical origin. The superior temporal gyrus and Heschl's gyrus have been identified as symptomatogenic zones in ictal deafness by mapping with electrocortical stimulation^14^ and resective surgery.^15^ Right hemifacial dysesthesia reflects seizure propagation to the left insular cortex or left parietal lobe. Based on the variety and sequence of ictal symptoms, we speculate that the seizures in our patient arose from the left claustrum‐insular‐operculum regions and propagated to the left lateral superior temporal and left parietal lobes or insula, subsequently involving the whole brain.

In conclusion, we propose that adult‐onset post‐FIRES epilepsy with startle‐induced seizures represents a largely unknown syndrome that may define a distinct subgroup of patients who occasionally develop startle‐induced seizures.

## CONFLICT OF INTEREST STATEMENT

None.

## CONSENT

Consent was obtained from the patient for publication of the report and associated images.

**Test yourself**

1. Which factor was unique in this study?
  - Adult‐onset startle epilepsy
  - Ictal deafness
  - Previous febrile infection‐related epilepsy syndrome
  - All of the above
2. Which EEG finding was characteristic during startle‐induced seizure in this study?
  - Normal EEG
  - Left temporal rhythmic delta activity, followed by rhythmic theta activity and repetitive sharp waves
  - Initial vertex discharge followed by diffuse relative flattening or low‐voltage rhythm
3. Which region was considered as the epileptogenic zone in this study?
  - Claustrum‐insular‐operculum
  - Mesial frontal
  - Mesial temporal

*Answers may be found in the* *supporting information*.

## Supporting information


Data S1.

## Data Availability

The data that support the findings of this study are available on request from the corresponding author. The data are not publicly available due to privacy or ethical restrictions.
