# Supplementary material for: Adult‐onset epilepsy with startle‐induced seizure after febrile infection‐related epilepsy syndrome: A case report
Source: Epileptic Disord. 2025 Apr 15;27(3):451–6. doi: 10.1002/epd2.70026 (PMC12203301; doi:10.1002/epd2.70026)
Supplement: Supplementary file 1 — Data S1. [file EPD2-27-451-s001.docx]

Answers

1. D.

2. B.

3. A.
